# Antibiotic Use on Goat Farms: An Investigation of Knowledge, Attitudes, and Behaviors of Missouri Goat Farmers

**DOI:** 10.3390/ani8110198

**Published:** 2018-11-06

**Authors:** Lauren K. Landfried, Ellen K. Barnidge, Patrick Pithua, Roger D. Lewis, Jonathan A. Jacoby, Christopher C. King, Carole R. Baskin

**Affiliations:** 1 Department of Nutrition and Dietetics, Doisy College of Health Sciences, Saint Louis University, 3437 Caroline St. Room 3076, St. Louis, MO 63104, USA; 2 Department of Behavioral Science and Health Education, College for Public Health and Social Justice, Saint Louis University, 3545 Lafayette Room 310, St. Louis, MO 63104, USA; Ellen.barnidge@slu.edu; 3 Department of Population Health Sciences, Virginia-Maryland College of Veterinary Medicine, Virginia Tech, 205 Duck Pond Dr. Room 372, Blacksburg, VA 24061, USA; ppithua@vt.edu; 4 Department of Environmental and Occupational Health, College for Public Health and Social Justice, Saint Louis University, 3545 Lafayette, St. Louis, MO 63104, USA; Roger.lewis@slu.edu (R.D.L.); Jon.Jacoby@aftonchemical.com (J.A.J.); Christopher.king@slu.edu (C.C.K.); 5 Department of Environmental Health and Safety, Washington University-St Louis, 4533 Clayton Ave, St. Louis, MO 63110, USA; carole.baskinslu@gmail.com

**Keywords:** antibiotic use, goat farmers, farming practices, antibiotic resistant bacteria, veterinarian

## Abstract

**Simple Summary:**

Overuse or inappropriate use of antibiotics in agriculture has been implicated in the development of antibiotic resistant bacteria, a significant and growing public health threat. In a previous study, we found that Missouri goats had a higher percentage of antibiotic residues at slaughter than predicted based on the national average, so we undertook this study to understand contributing factors. As farmers are typically the ones administering antibiotics to their animals, we set out to investigate Missouri goat farmers’ knowledge and attitudes regarding antibiotics, veterinarians, and antibiotic resistance using qualitative research interview methods. Our aims were to determine circumstances leading to farmers’ administration of antibiotics, farmers’ decision process resulting in the use of antibiotics, the role of veterinarians, and farmers’ perceptions about antibiotic resistance. The following themes emerged: how farmers detect illnesses in individual goats, herd health management, where farmers obtain antibiotics, and farmers’ thoughts about antibiotic resistance. Our findings highlighted the need for more emphasis on goat health management during veterinary education and the need for improved working relationships between veterinarians and farmers to promote appropriate antibiotic use and prevent the emergence of antibiotic resistant bacteria.

**Abstract:**

Use of low dose, prophylactic antibiotics contributes to the emergence of antibiotic resistant bacteria. In one study, goat meat in Missouri was found to have a higher percentage of antibiotic residues at slaughter than the national average, so we attempted to identify factors related to goat production that may contribute to this issue. Using the knowledge, attitude, and behavior (KAB) model, we interviewed 11 Missouri goat farmers about factors affecting antibiotic use. Most of the farmers did not have specific protocols for managing illnesses and only relied on veterinarians for major health issues. Many felt veterinarians lacked knowledge about goat medicine so instead relied on other farmers’ or their own experiences for treatment modalities. While most agreed that antibiotic resistance was a concern, only 4 of the 11 indicated that they only used antibiotics when prescribed by the veterinarian. Veterinarians should be relied on and valued for their medical expertise, but they are not always being utilized in this manner. Therefore, veterinary education should emphasize goat health management to a greater extent than it currently does, and soft skills to build collaborative relationships with farmers should be taught to promote preventative health measures and more judicious use of antibiotics.

## 1. Introduction

Animal production practices have been cited as major contributors to the emergence of antibiotic resistant bacteria, contributing to over two million illnesses and 23,000 deaths annually [1,2]. This is especially concerning because 70% of antibiotics used in animal production are considered medically important, or used in human medicine practices [3]. Although the European Union ban of the use of antibiotics as growth promoters did not affect Denmark’s profits from swine production [4], the perception that sub-therapeutic antibiotic administration is the best way to maximize health and growth of animals persists in the United States [4].

The beef, swine, and poultry industries have had increased scrutiny and oversight of antibiotic use over the years, but the goat industry has not received similar attention despite its increasing market share. According to the 2013 Red Book, which includes the results of the 2011 meat inspections performed by the United States Department of Agriculture’s (USDA) Food Safety Inspection Service (FSIS), goat meat was reported as having the highest percentage of drug residues compared with other types of meat [5]. These levels suggest that withdrawal times for drugs administered to goats are not always followed or are imprecise. Use of antibiotics in this species is predominantly extra-label, which means that dosages have to be extrapolated from other species by a veterinarian [6,7]. While the USDA increased their systematic sampling for species making up 95% of the meat market in 2012, this effort excluded goats [8]. In addition, Landfried et al. found a significantly higher amount of antimicrobial residue in goats raised in Missouri compared to what is being reported for goat meat nationally in the Red Book [9].

Veterinarians play an important role in preventing the overuse of antibiotics in goats. The Netherlands saw a 56% reduction in antimicrobial use for farm animals from 2007 to 2012 when it made efforts to support veterinary and farmer relationships, including setting up a task force comprised of veterinarians, farmers, and other stakeholders [10]. However, according to the 2013 report of the American Veterinary Medical Association (AVMA), food animal veterinarians were found to be underutilized with regards to preventative care [11]. This issue is not unique to the U.S., as sheep farmers in the U.K. indicated in a qualitative study that they felt veterinarians provide inconsistent service, have a high turnover, and lack expertise relevant to their herd [12]. Many of these farmers said they get recommendations from other farmers instead of veterinarians, which can lead to inappropriate drug use [12]. To support veterinary oversight of antibiotic use, the AVMA has established a Veterinarian-Client Patient Relationship (VCPR) to promote relationship building between producers and veterinarians as a step to ensure that treatment choices are evidence based [13]. To ensure best practices, the University of Wisconsin touts establishing a welfare team with the veterinarians at the lead as the best practice for beef production [14]. Therefore, all production farms should follow this recommendation for optimal animal wellbeing.

The knowledge, attitude and behavior (KAB) model suggests that behavior (in this case, administration of antibiotics) follows knowledge and attitudes [15]. For example, a belief that overusing antibiotics may ultimately harm a herd could result in more cautious use of antibiotics than the absence of such belief. In the past, the KAB model has been successfully applied to promote compliant use of medications [16] and even simple educational materials have resulted in behavioral changes, such as self-monitoring for stroke risk [17].

In the present study, we investigated farmers’ knowledge and attitudes about antibiotics, veterinarians, and antibiotic resistance with the goal of understanding their possible role in the burden of antibiotic-positive goat carcasses in Missouri. Our specific aims were to determine why farmers administered antibiotics to their herd, their decision process leading to the use of antibiotics, the role of their veterinarians, and farmers’ perceptions about antibiotic resistance.

## 2. Materials and Methods

### 2.1. Ethics and Experimental Design

This study was approved by the Institutional Review Board at Saint Louis University (protocol #27522). Goat farmers who consented to participate were interviewed using a focus group-tested questionnaire and interview guide, developed to capture (1) age, gender (2) flock size, (3) whether antibiotics were used and, subsequently, (4) reason using antibiotics, (5) role of veterinarian in the decision to use or not to use antibiotics and (6) their thoughts on antibiotic resistance.

### 2.2. Participant Selection

Goat farmers were identified using a Google search of goat farms in Missouri using the keywords “goat farms,” “goat farms in Missouri,” and “goat farmers in Missouri” in order to find the farms most interested in connecting with potential consumers using online mechanisms [18]. In addition, the website http://www.agrilicious.org/ was used to find farmers’ contact information in Missouri since it has a database of farmers who can be identified by state, species, and products sold [19]. Based on the searches, a total of 35 farms of the 3955 Missouri goat farms inventoried in the 2012 census, with an average of 26.2 goats (103,669/3955 = 26.2), were identified for recruitment [20]. Eligibility criteria to participate in the study included a working phone number and at least three goats on the farm. Farmers were assigned to six geographical regions of the state of Missouri based on their county to strive for representation of each region of the state in this study. Farmers were called in order of geographical region and were asked whether they could speak at that time or at a more convenient time. Once a region had a farmer who agreed to participate, a farmer from the next region was phoned. We interviewed farmers until we reached the qualitative research principle of data saturation, or when no new information was collected and no new themes or codes were able to be constructed to further add to the understanding of this study [21].

### 2.3. Farmer Interviews

We collected and analyzed data concurrently throughout this study [22]. The principle investigator conducted open-ended telephone interviews at a time convenient to farmers, ensuring their comfort. Farmers did not have to travel and the literature suggests that people tend to be at ease when an interview is conducted in their home [23]. The average interview time was 27 min with a range of 2.57 (for a farmer who did not use antibiotics) to 48.78 min.

Each farmer provided verbal consent to participate and responded to a demographic questionnaire. Farmers that answered ‘no’ to the last question of the questionnaire (indicating that they did not use antibiotics) were excluded from the study. 

The interview guide was then used to obtain the following information from farmers: health of the goats on their farm, protocols for diagnosing and treating medical conditions, types of antibiotics used, how they obtained medications, details about factors and criteria that lead them to decide to use antibiotics, who they rely on regarding overall animal health, the role of the veterinarian in administering antibiotics, whether farmers used extra-label antibiotics, their thoughts regarding antibiotic/antimicrobial resistance, and an open-ended question about anything else the farmer felt pertinent regarding their experience as a goat farmer. Some questions had additional prompts or follow-up questions for further exploration if needed. No additional questions were developed based on the data collected.

### 2.4. Data Analysis

The demographic questionnaires were used for descriptive analysis. The audio recordings were transcribed verbatim by a transcriptionist. All recordings were numbered to keep the identity of the farmer confidential. Transcripts were read along with recordings by the principal investigator to ensure accuracy of transcriptions. All data were de-identified for analysis.

Transcribed interviews and notes were analyzed qualitatively using grounded theory. The principle investigator, who also performed the interviews, used open coding to determine the main points of the data and selective coding for selecting specific themes. Another member of the research team experienced in qualitative research reviewed the transcripts and made suggestions based on some of the comments made by the interviewees. Comments were added to some codes, sub-codes were added to some of the codes, and some of the codes were omitted if they did not add value to the study.

## 3. Results

### 3.1. Demographics

A total of 35 farms were compiled based on the Google and Agrilicious searches, of which 5 did not have a working telephone number and had no other identifiable way to be reached, 13 did not return calls, 2 did not have any goats, 2 were emailed without a response, and 2 declined to participate. As shown in Table 1, a total of 12 farmers from 11 farms were interviewed for this study. The farmers represented the southern two-thirds of the state geographically as none in the northern-most third participated in the study. Farmers ranged in age from 30–79 years of age. All of them identified as white or Caucasian, with 3 indicating they had Bosnian, German, or German/Irish heritage. Goat herd numbers ranged from 9 to 120, and all the farmers had other animals on their farms. Meat and milk were the main products for most of the farms. After completing the demographic questionnaire, one of the farmers indicated that antibiotics had never been used for their goats. Therefore, only 11 farmers from 10 farms were fully interviewed for this project.

The following themes were captured among the interviews: identification of health issues in individual animals, herd health management, where farmers obtain antibiotics, and farmers’ thoughts about antibiotic resistance. Themes and subthemes are included in Table 2.

### 3.2. Themes from the Farmer Interviews

#### 3.2.1. Theme 1: Identification of Health Issues in Individual Goats

The first emerging theme found among the farmers was how they identify illnesses in goats. Most farmers did not have a specific protocol for determining the health of individual goats and any need for antibiotics. However, they felt confident in identifying illnesses. Several mentioned that they knew there was a problem with their goats when they would notice a change in their behavior, especially around meal time.

“Yeah, the last ones [goats] up to the feed dish. The ones that don’t run up to the feed dish. The last ones in the line up to the barn are the ones that get checked [for illnesses].”

(F5)

“We check the animals daily and if we have an animal that is not acting the way it should we do not carry. We check it over and narrow down the cause of the problem and treat accordingly.”

(F8)

The farmers stated that after they notice something awry with the goats, they further investigate to determine what the main problem is. One farmer mentioned that when the goats are acting unusual, he checks their ears, head, tail, temperature, fecal sample, and entire body. Another farmer did mention that he performs a 6-point binary check on the goats on a regular basis:

“We do a weekly check, like a 6-point binary check on the animals. We try to do it every week, sometimes it’s every 2 weeks, but we check basically the eyes for anemia and/or discharge. We check, well I sort of start at the other end, the tail, for scours. And then we check the coat condition, body condition, check the eyes, check the nose, and then check the jaw for any edema, so…it sounds lengthy but it’s a quick pass over on each animal.”

(F4)

#### 3.2.2. Theme 2: Herd Health Management

Another emerging theme found among the farmers was herd health management. The first subtheme identified was the role of the veterinarian in farm management and prescribing antibiotics. All the farmers stated that they had access to a veterinarian who could come to the farm and provide necessary treatment for their herds. However, it was noted by several farmers that they contact a veterinarian because of severe problems and not for minor infections. When asked to describe the role of the veterinarian on the farm, one farmer replied:

“Not much, I mean truthfully… When I call he knows it must be something horrible, a blood transfusion or something…I don’t call him every time I think something needs an antibiotic, no.”

(F5)

Cost was a subsequent subtheme that emerged from the interviews. Several farmers noted that service costs were a barrier for getting the veterinarian to the farm.

“Um…it generally costs nearly as much as a goat’s worth to call in a veterinarian out here, so unless I’ve got a problem with the herd overall, then it would be a zero.”

(F3)

One farmer did mention, however, that cost would never prevent getting appropriate care for the goat. This farmer mentioned that she is a member of an animal welfare approved program, so understands the importance of the veterinarian being involved in the treatment of the animals.

Trust of veterinarians was another subtheme that came up. Overall the farmers noted that they trust their veterinarian. Most farmers felt that they could trust their veterinarian’s recommendations with regards to antibiotic use, although most veterinarians are not always contacted before administration of antibiotics.

“Uh, yeah…the way I would phrase that is I trust her to do anything structural, or you know, that she would have to do to get in there and save the animals life, but I don’t think that they have the information on goat care. I really like our veterinarian but I don’t really think she believes that dewormer resistance is a problem, so…”

(F4)

“Int: Do you trust your veterinarian’s recommendations?

F5: Certainly.

Int: So you use antibiotics…[when prescribed by a veterinarian]?

F5: I combine it, but I combine it with my experience as well, because I have more experience, you know, but he [the veterinarian] has sometimes some really good ideas or alternative thoughts, you know, which, well part of our relationship is a good give and take.”

(F5)

Another subtheme that emerged during interviews was farmers’ perception of how knowledgeable veterinarians were about goat herds compared to farmers. While farmers felt that they could trust veterinarians in regards to antibiotic recommendations, the majority did not feel that veterinarians had as much knowledge regarding goats as farmers do. Therefore, farmers felt that they could rely on their own knowledge and experience regarding treatment of their goats rather than reach out to the veterinarian. Regarding overall herd health, one farmer said that she relies on herself more than on the veterinarian:

“That would probably just be myself. I use one of the vets a little bit. There’s not a lot of veterinarians that have a lot of knowledge about goats in our area, so it’s really hard to have a vet by your side to work with you on your farm and prescribe medications accordingly as I see fit for my goats. So, I think mostly I do most of the intervention for my farm… I’m not saying that I know more than a veterinarian because that’s not true at all, but I think I’m able to better care for them in my point of view than what they can help me with as of right now.”

(F10)

In addition, some farmers relied on other farmers and social media, such as Facebook groups where veterinarians respond to general questions, for their antibiotic recommendations rather than seeking out traditional veterinary care.

When it comes to specific knowledge of drug withdrawal times, which is the minimum time allowed between the last dose of a medication being administered and slaughter or milking, 3 of the farmers indicated that they felt comfortable in their knowledge.

“Well because we’re a participant of animal welfare approved program, which is now a greener world. We have to log every time we use antibiotics, in a book, with the date and the lot number. We have to double the amount of time for withdrawal period should we decide to slaughter that animal. So we just, you know, it’s all in the book.’’

(F9)

“Int: Can you describe some disadvantages [of using antibiotics]?

F7: We only use them for sick animals. Yeah, disadvantages is withdrawal time. Obviously we have to destroy milk for a period.” (F7)

While farmers do not feel that their veterinarian is always knowledgeable about goats, 4 of the 10 farmers indicated they only use drugs prescribed by the veterinarian. The other farmers were not concerned about using drugs not prescribed by a veterinarian.

“You know, I’m not going to say a situation couldn’t come up [where I would use antibiotics not prescribed by a veterinarian], but I can’t think of any other reason, other than a large wound, I can’t think of any other reasons I would be using them [antibiotics].”

(F3)

Several indicated that they have certain antibiotics on hand to use on a case by case basis.

“The only two I really use is Pen-G and Oxytetracycline and I keep those on hand in the refrigerator, when you need them you need them. Pen G is a good antibiotic. It’s good for gram negative, gram positive, and it works on 99% of everything. If I don’t get a good response with the Pen G then I’m calling my vet.”

(F6)

When probed further about their use of antibiotics without a prescription, some farmers indicated they chose the particular antibiotics based on their own experiences. A couple even indicated no concerns when using antibiotics in this manner given their personal expertise in their herd management:

“No, I don’t really have any concerns. I’ve used them [antibiotics] in the past, and I feel that my knowledge about them [antibiotics] is pretty good.”

(F11)

Speed of the illness progressing was mentioned by a couple of the farmers as a reason for administering the antibiotics before getting a veterinarian consult.

“Well I mean if it has to be a veterinarian to give me the medication, like Nuflor and stuff like that, I’ve gotten bottles of that to use on my farm, so I guess it would be prescribed. They prescribe that to my farm, for me to use on my farm, I can’t just get that at a feed store or anything like that. It’s a little more stronger antibiotic, a little more aggressive than Penicillin, especially when there’s a goat with mastitis or a serious respiratory, you really need to act fast on those illnesses that you don’t have a lot of time with.”

(F11)

All of the farmers indicated they only medicated the ill goat or goats.

#### 3.2.3. Theme 3: Where Farmers Obtain Antibiotics 

While many farmers interviewed relied on veterinarians for the specific drugs used on their goats, they also felt confident in getting antibiotics at a feed store or online. Only 4 of the 8 farmers indicated that they only use drugs prescribed by veterinarians.

“F6: I order some through the mail and feed store and the vet.

Int: Do you need a prescription for the ordering online or feed store?

F6: No, I don’t get prescription antibiotics, I just get them at the feed store, because you can, so I just go to the vet and get any medicines that I can’t pick up on my own.”

(F6)

#### 3.2.4. Theme 4: Thoughts about Antibiotic Resistance

The final emerging theme found among the farmers was their attitudes regarding antibiotic resistance. When asked about the disadvantages of antibiotic use, one farmer indicated that resistance was his biggest fear. All of them feel that antibiotic resistance is a problem and that farmers may play a role in the emergence of resistant bacteria.

“I think they [farmers] do. Yeah, the last line, we’re farmers and the ones who actually administer the antibiotics, I do think that they do play a huge role in it.”

(F7)

Many indicated that they felt that the larger farms may have a harder time managing their goats’ health without using antibiotics.

“Now there are some big-ticket operations and I’m sure, you know, that’s a whole different dynamic. That’s, they couldn’t, probably couldn’t function, couldn’t keep up their management practice without it [antibiotic administration].”

(F5)

## 4. Discussion

Since antibiotic resistant bacteria are becoming an increasing concern with livestock, it is prudent to use antibiotics only when necessary [3,24,25]. This is the first study looking into knowledge and attitude of goat farmers with regards to antibiotic use in the United States. Using the KAB model, the findings of this study reflect how knowledge and attitudes may play a role in the administration of antibiotics in goat farms in Missouri, and subsequently how resulting practices contribute to development of antibiotic resistance. The themes emerging from the interviews show how decisions regarding antibiotic use are made.

Identification of illnesses in individual animals was the first theme that emerged. Only one farmer had a specific protocol for determining the health and well-being of his animals. All the same, farmers felt that they had intimate knowledge of their goats’ behaviors and the ability to identify abnormalities that may indicate an illness: lack of appetite (as evidenced by being last to the food dish), limping, and any other visible problems. Farmers indicated that they felt comfortable acting independently on this knowledge. While all of the farmers regarded their goats as healthy and felt that they had a good understanding of herd health, protocols to assess health may help in preventing infections and thus reduce the use of antibiotics [3]. A formalized checklist to assess health and wellness of animals kept on the farm may be of benefit.

While all farmers had access to a veterinarian, they tended to rely on the veterinarian only for major health issues. This is consistent with the USDA National Animal Health Monitoring System (NAHMS) Goat 2009 study that found only a third of farming operations had consulted a veterinarian in the last year [26]. Cost was mentioned as a barrier for some of the farmers interviewed, which was also determined to be a barrier among other small ruminant farmers [12]. Only one of the farmers indicated that cost of veterinary care was not a barrier when deciding to use antibiotics. This farmer also mentioned that they were a participant in an animal welfare approved program, so her values (attitude) about the importance of veterinary care to ensure welfare of the herd may play a role in this decision. This is not unique to goats, as cost has been cited as a barrier for sheep and even cattle farmers seeking veterinary care [12,27,28]. In addition, speed of the illness progressing was mentioned as a barrier for not seeking out veterinary assistance. This indicates that the veterinarian may not be as responsive to the issues the farmer perceives important. All of this suggests that farmers must feel that the veterinarian is a valuable asset to their goat health management in order to utilize them to the fullest.

The biggest concern that emerged from the interviews, though, was the lack of knowledge or perceived knowledge of the veterinarians about goat medicine. This may seem a reasonable concern, as in the United States there is limited time spent in veterinary school focusing on small ruminants [29]. Another complication is that many medications for small ruminants, especially goats, are extra-label, so dosage and withdrawal times have to be fully understood [29]. In addition, many veterinarians that care for small ruminants see companion animals or horses as their primary practice [29]. Therefore, many have to seek out additional continuing education opportunities such as membership with the American Association of Small Ruminant Practitioners (AASRP) or continuing education to properly care for goats [29]. Research by Landfried et al. [30] found that of the respondents, Missouri veterinarians actually had less experience than veterinarians from other states about small ruminant animals. While education is important, Missouri veterinarians have among the lowest continuing education requirements of all U.S. states [31,32], which is surprising considering that Missouri ranks third in total number of goats in the U.S. [20]. Therefore, Missouri veterinarians may not be taking advantage of all the educational opportunities and perhaps may have less knowledge than the farmers would hope. When asked about trust in their veterinarian, however, all but one of the farmers said they do trust their veterinarians’ recommendations within reason. In the context of a growing goat meat industry, all of this suggests the need for better understanding of goats among veterinarians as well as a better relationship between farmers and veterinarians [33].

Due to perceived lack of knowledge of their veterinarian, many of the farmers indicated that they relied either on their own experiences, Facebook groups (which include veterinarians who respond to general questions), or other farmers for the care of their goats as well as their information regarding antibiotic use. In particular, many of the farmers stated that they felt they had the knowledge needed to identify the illnesses, understood how they needed to treat problems, and felt confident in their usage of antibiotics based on the instructions on the bottle and prior experiences. This is consistent with research from Kaler and Green [12], where they found similar results from sheep farmers in the United Kingdom. While reliance on personal experience is important, the NAHMS Goat 2009 study found that goat farmers, especially those that farmed for meat, had little knowledge about antibiotics and withdrawal times [26]. This is concerning in the context of Landfried et al.’s findings [9] regarding high levels of antibiotic residues in Missouri goat kidneys. While the farmers indicated that they only medicated the goats of concern, administering antibiotics without veterinary oversight could put these goats at risk for overuse of these drugs.

An emerging theme coming from the interviews was where the farmers were able to get antibiotics. Even though the United States Food and Drug Administration (FDA) has repeatedly recommended that veterinarians be involved in all antibiotic prescription and administration protocols, most antimicrobial drugs can be easily purchased over the counter [25]. Only four of the farmers indicated that they only use drugs prescribed by veterinarians. The ease of purchasing antibiotics at feed stores and online may contribute to the emergence of antibiotic resistant bacteria [34].

Most of the farmers interviewed felt that antibiotic resistance was a problem to which farmers contributed. However, the interviewees also felt that they did not use antibiotics very much compared to larger farms (over 200 goats). The farmers felt that their ability to care for their herd more closely and their low use of antibiotics prevented the emergence of antibiotic resistance on their own farms. To ensure antibiotic usage is low, the aforementioned formalized herd health checklist could help.

This study helped ascertain some of the reasons for use of antibiotics by goat farmers. The KAB model pointed to two areas to address: knowledge of antibiotic use (both dosages and withdrawal times) for farmers as well as veterinarians (with regards to their own educational requirements) and attitude towards veterinarians. Overall, they felt they had good knowledge of the health of their goats. They also felt confident in their knowledge in the antibiotics used on their farms. While the farmers interviewed said that they trusted veterinarian recommendations, they did not feel it always necessary to contact the veterinarian when a problem arose, indicating that their behavior is not congruent with their attitude. There is a consensus among veterinarians that the information provided about small ruminant husbandry and medicine in veterinary schools is limited [29]. Therefore, increased education in this field, especially with regards to extra-label drugs, should be an option for those practicing in rural areas. Veterinarians having more knowledge about goat farming should help improve the attitudes of farmers toward their recommendations, allowing farmers to appreciate and value veterinarians during times of need. In addition, a better working relationship with the veterinarians in the forms of preventative care may lessen the need for antibiotics used on the farm.

While this study discovered valuable findings, there were several limitations. The sample size was small and did not include large-scale producers. The farmers interviewed indicated that large-scale operations were most likely to misuse antibiotics, as has been shown for other species [35,36], so their input would have been helpful to get a better view of the overall problem. In addition, we used farms that advertised online for our potential participant pool, since farmer organizations would not release membership information. This may have biased our study toward less-established farms that need online advertising to find customers. Finally, in the context of national attention to proper usage of antibiotics, this study design lends itself to the possibility of social desirability bias [37]. However, these limitations do not diminish our finding of the critical nature of a collaborative veterinarian-producer relationship to encourage judicious use of antibiotics on the farm.

## 5. Conclusions

Overall, the interview process was helpful in determining farmers’ feelings towards antibiotic use, about veterinarians, and the role of goat farming in the emergence of antibiotic resistant bacteria. Veterinarians should be the experts for farmers to call on to prevent and treat illnesses. Therefore, education about goat farming and tools for relationship building with farmers should be covered in veterinary school and continuing education courses. A productive client–veterinary relationship can boost preventative care and educate the farmers on best practices to reduce harmful use of antibiotics for minor problems. In addition, antibiotics should not be available for purchase over the counter, as per current recommendations by the FDA. In the future, we hope to get the veterinarians’ perspective on their role in the combat against the emergence of resistant organism by food animal production facilities.

## Figures and Tables

**Table 1:** Information about farmers: demographics, goat farming characteristics, other animals on farm, and occupation.

Farm	Age of Farmer	Gender	Number of Goats	Length of Time Farming Goats	Main Products	Full-Time Goat Farmer (Yes/No)	Antibiotic Use (Yes/No)
1	36	M	20	5 years	Milk–to make soaps, lotions, and other products	no	Yes
2	53	M	100–120 (usually 100–200)	20 years	Meat and other (breeding stock)	no	Yes
3	35	M	24–25	4 years	Meat	no	Yes
4	45	M	101	4 years	Meat	yes	Yes
5	65	F	30	45 years	Wool, milk, meat	Retired	Yes
6	79	F	25–30	54 years	Meat and milk	Retired	Yes
7	52	F	79	10 years (6 commercially)	Milk-goat cheese	yes	Yes
8	55	F	30	12–13 years	Meat and other (breeding stock)	no	Yes
9	3330	FF	20	3–4 months	Milk and other (soap)	yes, but also has a part-time job	Yes
10	37	F	60–65	9 years	Milk and meat	no	Yes
11	50	F	9	5 years	Meat, wool/fiber, milk, and other (soap)	yes	No

**Table 2:** Themes and subthemes that emerged from farmer interviews regarding their use of antibiotics.

Themes	Subthemes (If Applicable)
Identification of health issues in individual animals	
Herd health management	Role of the veterinarian
	Cost
	Trust of veterinarian
	Perceived knowledge of veterinarian/farmers
	Other farmers and social media
	Use of drugs when prescribed by veterinarian
Where farmers obtain antibiotics	
Thoughts about antibiotic resistance	Farmers play a role
	Larger farms

## Supplementary Materials

Supplementary File 1
