# Supplementary material for: Antibiotic Use on Goat Farms: An Investigation of Knowledge, Attitudes, and Behaviors of Missouri Goat Farmers
Source: Animals (Basel). 2018 Nov 6;8(11):198. doi: 10.3390/ani8110198 (PMC6262384; doi:10.3390/ani8110198)
Supplement: Supplementary File 1 [file animals-08-00198-s001.zip › Approved_Demographic Questionnaire.pdf]

Demographic Questionnaire

How old are you?: ______________________

How would you describe your race or ethnicity?: ________________________________

Address: ______________________________

____

Address of goats (if different): ______________________________

____

How many goats are on your farm?: ________________________
- (if less than 3, then end the survey)

How long in years have you been practicing as a goat farmer?: _________________________

Other than goats, what other animals are on your farm?:
____

Check the types of farming practices/products your farm produces:
- ☐ Meat
- ☐ Milk
- ☐ Wool/Fiber
- ☐ Other

Is farming your full-time occupation? _________________________

If no, what is: ______________________________________

Have you ever used antibiotics for your goats? ____
- (If no, end here. If yes, go on to the interview)
