# Supplementary material for: Antibiotic Use on Goat Farms: An Investigation of Knowledge, Attitudes, and Behaviors of Missouri Goat Farmers
Source: Animals (Basel). 2018 Nov 6;8(11):198. doi: 10.3390/ani8110198 (PMC6262384; doi:10.3390/ani8110198)
Supplement: Supplementary File 1 [file animals-08-00198-s001.zip › Approved_Interview Guide.pdf]

Broad purpose of the study is to determine the farmers' use of antibiotics.

Specific aims:

1. Why do they use antibiotics with their goats?
2. When do they know they need to use antibiotics?
3. What role does a veterinarian play in their decision to use antibiotics?
4. What types of antibiotics do they use for what conditions?

Interview Guide

1. How would you describe the health of the goats on your farm?
2. Do you have a protocol for diagnosing and treating medical conditions on your farm?
    a. If yes – could you please describe your protocol to me?
    b. If no – why do you not have a protocol?
3. Who do you rely on regarding overall animal health?
    a. Where do you get most of your information regarding antibiotics?
4. When your goats are sick, what types of antibiotics do you use on your goats?
    a. What conditions do you treat with antibiotics?
    b. How do you get these medications?
5. Can you tell me what factors/criteria lead you to decide to use antibiotics on your goats?
    a. Can you tell me some benefits of using antibiotics on your animals?
    b. Can you describe some disadvantages to using antibiotics on your animals?
    c. Do you use any alternative treatment methods for your goats?
        i. Why/why not?
6. What role does a veterinarian play in your decision to administer antibiotics?
    a. Can you describe the relationship you have with your goats' veterinarian?
    b. Does the veterinarian come to the farm or do you have to bring your goats to him/her?
    c. Does your veterinarian do any phone consulting?
        i. If yes – could you describe the circumstances?
    d. Do you trust the veterinarian's recommendations?
        i. Why/why not?
7. Do you use antibiotics not prescribed by a veterinarian on your farm?
    a. If so, what kinds?
    b. Are there any other considerations when using antibiotics in this manner?
    c. Is cost a factor when deciding to use antibiotics not prescribed by a veterinarian?
8. What are your thoughts about antibiotic resistance?
9. Do you feel that farmers play a role in antibiotic/antimicrobial resistance?
    a. Please explain
10. Is there any other information that you feel is important for me to know about your experience as a goat farmer?
