# Supplementary material for: Antibiotic Use on Goat Farms: An Investigation of Knowledge, Attitudes, and Behaviors of Missouri Goat Farmers
Source: Animals (Basel). 2018 Nov 6;8(11):198. doi: 10.3390/ani8110198 (PMC6262384; doi:10.3390/ani8110198)
Supplement: Supplementary File 1 [file animals-08-00198-s001.zip › Table of Themes, Subthemes, and Supportive Quotes from Farmer Interviews.docx]

Table 2: Themes, subthemes, and supportive quotes from the farmer interviews

| **Theme** | **Subtheme (if applicable)** | **Quotes to support the theme** |
| --- | --- | --- |
| Identification of problems with goats |  | “Yeah, the last ones up to the feed dish. The ones that don’t run up to the feed dish. The last ones in the line up to the barn are the ones that get checked [for illness].” (F5) |
|  |  | “We check the animals daily and if we have an animal that is not acting the way it should we do not carry. We check it over and narrow down the cause of the problem and treat accordingly.” (F8) |
|  |  | “Um, I think most of it is that I’m pretty familiar with each individual animal. I know how they act, and I, intervention accordingly as necessary according to their needs. I don’t do a test or anything like that if that’s what you’re talking about.” (F10) |
|  |  | “They are not pets, but yeah, I do keep an eye on them. I do run around and check them at least every few days, but they are so very trouble free, the ones I’m raising. I’ll give you a run down of my expenditures, other than fence, but for the last year. One large bale of hay, two sacks of sweet feed, one sack of mineral, part of a sack of kelp, and one bucket of protein, protein lick for the winter.” (F3) |
|  |  | “We do a weekly check, like a 6 point binary check on the animals. We try to do it every week, sometimes it’s every 2 weeks, but we check basically the eyes for anemia and/or discharge. We check, well I sort of start at the other end, the tail, for scours. And then we check the coat condition, body condition, check the eyes, check the nose, and then check the jaw for any edema, so…it sounds lengthy but it’s a quick passover on each animal.” (F4) |
| Goat health management | Role of the veterinarian | “Not much, I mean truthfully. I don’t see him that often. When I call he knows it must be something horrible, a blood transfusion or something. You know he hates it when I call, oh my gosh, what happened?! Most things I can manage here on the farm. If it’s something that I know I cannot manage here then I certainly call upon him. You know, we have a good relationship. I don’t call him every time I think something needs an antibiotic, no.” (F5) |
|  | Cost | “Um…it generally costs nearly as much as a goats worth to call in a veterinarian out here, so unless I’ve got a problem with the herd overall, then it would be a zero.” (F3) |
|  |  | “Never. I would not, no. I would not put an animals need before my, no, not like that….Only if the cost of, if a goat did something and it’s leg is hanging off, and it’s like yeah, I can take it to the vet or I can just euthanize it, what is better in the long run, I have gone to euthanasia, yes.” (F8) |
|  | Trust of veterinarians | “Uh, yeah…the way I would phrase that is I trust her to do anything structural, or you know, that she would have to do to get in there and save the animals life, but I don’t’ think that they have the information on goat care. I really like our veterinarian but I don’t’ really think she believes that dewormer resistance is a problem, so…” (F4) |
|  |  | “Int: Do you trust your veterinarians recommendations?  F5: Certainly.  Int: So you use antibiotics…[when prescribed by a veterinarian]?  F5: I combine it, but I combine it with my experience as well, because I have more experience, you know, but he has sometimes some really good ideas or alternative thoughts, you know, which, well part of our relationship is a good give and take.” (F5) |
|  |  | “Oh, only when they make sense, because I realize that they’re highly trained, but they are just people too. So in order to maintain your goats really, really well, it has to jive with everything, you know? If they think it’s something rare, like if they think your goat aborted because it had lepto or something like that and none of your other goats had the symptoms, and the only symptom is abortion, it’s quite possibly something more simply like soybean meal.” (F9) |
|  | Perceived knowledge of veterinarian/farmer  Perceived knowledge of farmer | “That would probably just be myself. I use one of the vets a little bit. There’s not a lot of veterinarians that have a lot of knowledge about goats in our area, so it’s really hard to have a vet by your side to work with you on your farm, and prescribe medications accordingly as I see fit for my goats. So I think mostly I do most of the intervention for my farm.” (F10) |
|  |  | “We work pretty closely with a vet in the area. When we first started using her she told us I don’t know much about goats but I’m willing to learn, and I think that’s true for a lot of vets. Someone had told me, I think it’s another vet that’s in her office, that she just came to the practice recently that they spend a half a day on goats in vet school.” (F7) |
|  |  | “We sell for very good money, and I mean I know these animals every day. I know how they act every day, and if I feel that, I’m not going to take what the vet says over my knowledge of what I see every day in these animals, so that would be very hard for me. Now if I could have a very good relationship with a veterinarian and we had a trusting relationship it would be a little bit different, but I have not gotten that yet with a veterinarian, and I think it’s good because I’ve grown a lot of knowledge in almost the ten years that I’ve been raising these animals. I’m not saying that I know more than a veterinarian because that’s not true at all, but I think I’m able to better care for them in my point of view than what they can help me with as of right now.” (F10) |
|  | Withdrawal times | “Int: Can you describe some disadvantages?  F7: We only use them for sick animals. Yeah, disadvantages is withdrawal time. Obviously we have to destroy milk for a period.” (F7) |
|  |  | “Well because we’re a participant of animal welfare approved program, which is now a greener world. We have to log every time we use antibiotics, in a book, with the date and the lot number. We have to double the amount of time for withdrawal period should we decide to slaughter that animal. So we just, you know, it’s all in the book.“ (F9) |
|  | Other farmers and social media | “Other people in the business mostly. I also consult my vet when I have heard of some effective treatment for some particular thing that might be going through the herd or what seems to be going through other people’s herds. I’ll hear about it from them and then I’ll talk to my vet about it.” (F2) |
|  |  | “Then I also belong to, it’s a Facebook page that I try to go to them where you can post pictures if you’re having a goat medical issue and they’ll, they have registered vets that will respond to you, and they have a rule that if you’re just a member you can’t respond to a post, so it’s just the veterinarians that respond to the posts. So that’s a good resource as well.” (F1) |
|  | Use of drugs when prescribed by veterinarian | “The only times we’ve had to use them [antibiotics] was with the veterinarian.” (F1) |
|  |  | “You know, I’m not going to say a situation couldn’t come up [where I would use antibiotics not prescribed by a veterinarian], but I can’t think of any other reason, other than a large wound, I can’t think of any other reasons I would be using them [antibiotics].” (F3) |
| Where they get their antibiotics |  | “….the vet came out and she told us that’s what we needed to do so we got it, I think it was either at the farm store or NFA, and treated her [goat] pretty heavy for a couple days and then she came back and is fine now.” (F1) |
|  |  | “F6: I order some through the mail and feed store and the vet.  Int: Do you need a prescription for the ordering online or feed store?  F6: No, I don’t get prescription antibiotics, I just get them at the feed store, because you can, so I just go to the vet and get any medicines that I can’t pick up on my own.” (F6) |
| Thoughts about antibiotic resistance | Farmers play a role | “I think they do. Yeah, the last line , we’re farmers and the ones who actually administer the antibiotics, I do think that they do play a huge role in it.” (F7) |
|  |  | “I think that’s kind of what I’m saying earlier about giving them too much, too frequently. I just feel like, just like with people, you try not to, my mom was an RN, and she told me all the years growing up there’s some things you just need to let run their course instead of pushing antibiotics to get through it sooner. Sometimes things just need to run their course and let your body get through them. I think it’s easier for my wife and I, as adults to do that, then always expect the kids to go through, especially at a young age, but yeah, I think there’s definitely benefit to letting, kind of doing what I do as far as separating and just monitoring their intake of water and nutrition and things like that, and putting them in an environment where they’re more comfortable and less stressed, and let the body run its course. Your body or your animals, goats, can definitely, giving antibiotics too frequently can build up resistance to it.” (F1) |
|  |  | “You know, that’s very similar to antibiotic issues. You know, from use, regular use, you know, you breed for resistant varieties or resistant bacteria or resistant parasites. You know, you’re not only farming goats, but you’re also farming you know, resistant parasites. Same way with the sheep, but the goats are a little more sensitive to them than sheep or cows are, quite a bit more actually. They’re my canaries in the coal mine. You know, if I see, and I have angora goats as well, and they’re the worst of all of them, the most susceptible.” (F5) |
|  | Larger farms | “Most farmers I know, no. I live in an area where there’s lots of small farms, not big corporate farms. Now there are some big ticket operations and I’m sure, you know, that’s a whole different dynamic. That’s, they couldn’t, probably couldn’t function, couldn’t keep up their management practice without it. (F5) |
|  |  | “Oh yeah. I mean ultimately you know, the herd is my responsibility and their care and what they take in, it’s set on me, so I think I play a complete role on how much, how often, when, and it’s just my opinion, but I think the larger of the farmer is as far as herd size and things like that, whereas in my situation, where these are basically pets, I mean I can tell you each of their names and personality, I think the larger, if I was having a herd of 200 that it starts becoming, or it could be, I can’t say, but I can see where it becomes less personable, easier to do what you’ve got to do to get them better and don’t have time to separate each and every one of them. I think it definitely, the farmer definitely plays a role in how they’re treated and what they’re given and their care.” (F1) |
